# Cold Atmospheric Plasma Induces Apoptosis and Oxidative Stress Pathway Regulation in T-Lymphoblastoid Leukemia Cells

**DOI:** 10.1155/2017/4271065

**Published:** 2017-08-29

**Authors:** Eleonora Turrini, Romolo Laurita, Augusto Stancampiano, Elena Catanzaro, Cinzia Calcabrini, Francesca Maffei, Matteo Gherardi, Vittorio Colombo, Carmela Fimognari

**Affiliations:** ^1^Department for Life Quality Studies, Alma Mater Studiorum-Università di Bologna, C.so D'Augusto 237, 47921 Rimini, Italy; ^2^Department of Industrial Engineering, Alma Mater Studiorum-Università di Bologna, Via Saragozza 8, 40123 Bologna, Italy

## Abstract

Cold atmospheric plasma (CAP) has shown its antitumor activity in both *in vitro* and *in vivo* systems. However, the mechanisms at the basis of CAP-cell interaction are not yet completely understood. The aim of this study is to investigate CAP proapoptotic effect and identify some of the molecular mechanisms triggered by CAP in human T-lymphoblastoid leukemia cells. CAP treatment was performed by means of a wand electrode DBD source driven by nanosecond high-voltage pulses under different operating conditions. The biological endpoints were assessed through flow cytometry and real-time PCR. CAP caused apoptosis in Jurkat cells mediated by p53 upregulation. To test the involvement of intrinsic and/or extrinsic pathway, the expression of Bax/Bcl-2 and caspase-8 was analyzed. The activation of caspase-8 and the upregulation of Bax and Bcl-2 were observed. Moreover, CAP treatment increased ROS intracellular level. The situation reverts after a longer time of treatment. This is probably due to compensatory cellular mechanisms such as the posttranscriptional upregulation of SOD1, CAT, and GSR2. According to ROS increase, CAP induced a significant increase in DNA damage at all treatment conditions. In conclusion, our results provide a deeper understanding of CAP potential in the oncological field and pose the basis for the evaluation of its toxicological profile.

## 1. Introduction

Previous researches have repeatedly proven the anticancer effects of cold atmospheric plasmas (CAPs). Plasma effect on cancer cells is mediated by biologically active factors such as electric field [1–3], charged particles (ions and electrons), photons and UV radiations, free radicals, and reactive oxygen and nitrogen species (RONS) [4], including atomic oxygen (O), hydroxyl radical (·OH), superoxide (O_2_^−^), hydrogen peroxide (H_2_O_2_), atomic nitrogen (N), and nitric oxide (NO), generated both in gas and liquid phases [5]. Moreover, this blend of reactive species plays a major role in the induction of apoptosis in cancer cells directly exposed to CAP [6–9] or indirectly treated by means of plasma-activated liquids [10–18]. In particular, plasma treatment of complete cell medium induces the increase of extracellular RONS concentration [19] and this plays a crucial role in the effects of CAP on cells [20]. The anticancer effects mediated by reactive species are imputable to biochemical changes induced in the cells by the gas phase RONS and liquid phase RONS products [21]. Different studies demonstrated that RONS generated by plasma treatment can trigger cell signaling pathways involving JNK and p38 [22] and p53 [23], thus, promoting mitochondrial perturbation and activation of caspases [8], finally leading to apoptosis. The alteration of redox signaling induced by CAP treatment correlates not only with the induction of apoptosis but also with DNA damage [24], via DNA strand break formation and consequent activation of DNA damage checkpoints [8]. The antitumor effect of CAP was also explored on *in vivo* models. Vandamme et al. [25] evaluated the potential antitumor effect of an *in vivo* plasma treatment on a U87-luc glioma tumor xenograft, showing a significant reduction of tumor mass after 5 days of plasma treatment. Likewise, Chernets et al. demonstrated that plasma induced tumor suppression of subdermal melanoma in mouse model, via the increase of RONS levels [7]. Furthermore, a significant inhibition of tumor growth in *in vivo* model was shown by plasma-activated Ringer's lactate solution [12] and plasma-activated cell culture medium [26, 27]. Several studies demonstrated that the antitumor effect of plasma-activated liquids can be attributable to RONS and to the activation of solution component (e.g., L-sodium lactate) [12].

Clinical applications of plasma in the treatment of tumor are still missing, but a recent work of Metelmann et al. [28] reported a superficial partial remission of tumor on patients afflicted with advanced squamous cell carcinoma after CAP treatment. Recent papers proposed CAP as a promising anticancer strategy not only for its cytotoxic potential but also for its ability to simultaneously activate the immune system against cancer, which in turn determines the long-term success of anticancer therapy system [29]. In fact, redox molecules, such as, NO and ROS, and redox chemistry have a key role as immunomodulators in tumor and pathogen killing [30]. The optimization of plasma parameters would allow the induction of immunogenic cell death in tumors locally that will trigger a specific and protective immune response systematically [31].

Thus, plasma could be proposed as an interesting anticancer treatment, but it is necessary to deepen the understanding on the mechanisms and the specific components of CAP responsible of its anticancer effects. In this study, we investigated the proapoptotic effect of CAP and its ability to modulate the oxidative stress pathway in human T-lymphoblastoid leukemia cells (Jurkat cells) and identified some of the molecular mechanisms triggered by CAP treatment. We demonstrated that the exposure of complete medium to CAP produced by a nanosecond-pulsed DBD induced the generation of several RONS; among these species, nitrites and hydrogen peroxide are considered the most significant RONS contributing to plasma toxicity on cancer cells.

## 2. Materials and Methods

### 2.1. Cell Culture

Jurkat cells were purchased from LGC Standards (Teddington, UK) and cultured in RPMI 1640 supplemented with 10% heat-inactivated fetal calf serum, 1% antibiotics [penicillin 5000 IU/streptomycin 5 mg/mL], and 1% L-glutamine solution (all purchased from Biochrom, Billerica, MA, USA). Cultured cells were maintained in 5% CO_2_ and humidified air at 37°C.

### 2.2. Detection of Hydrogen Peroxides and Nitrites in Plasma-Treated Medium

The Amplex® Red Hydrogen Peroxide Assay Kit (Thermo Fisher Scientific, Waltham, MA, USA) and Nitrate/Nitrite Colorimetric Assay (Roche, Basel, Switzerland) were used according to the manufacturer's protocol to measure the concentrations of hydrogen peroxide and nitrites induced by plasma treatment in 1 mL complete cell culture medium. Plasma-treated medium was diluted 100-fold in PBS immediately after treatment, to obtain a solution with hydrogen peroxide concentration below 10 *μ*M and avoid any influence of pH on the measurement. The absorbances were measured photometrically with a microplate reader (Rayto, Shenzhen, P.R. China).

### 2.3. Cell Treatment with CAP

5 × 10^5^ cells in 1 mL complete medium were seeded in a monolayer through centrifugation and directly exposed to plasma treatment in a 24-well plate. The adopted plasma source is a nanosecond-pulsed dielectric barrier discharge (DBD) consisting of a cylindrical internal brass electrode covered by a glass test tube with a semispherical tip as dielectric (1 mm thick) [32]. Two operating conditions were selected following preliminary experiments, as already reported by the authors [11]; the first one consists in a 60″ treatment keeping a distance of 1.25 mm between the tip of plasma source and the surface of the cell medium (gap) (T1); while the second treatment condition consisted in a 120″ treatment performed setting the gap at 2.50 mm (T2). For each of the two operating conditions, preliminary experiments were performed, demonstrating that for the achievement of similar cellular effects, it is necessary to increase the exposure times when using greater distances and reduce them when using smaller distances [11]. During treatment time, peak voltage (PV) and pulse repetition frequency (PRF) were kept constant at 20 kV and 500 Hz, respectively.

### 2.4. Analysis of Cell Death Mechanisms

After treatment, aliquots of 2.0 × 10^4^ cells were stained with Guava Nexin Reagent (100 *μ*L) (Merck Millipore, Billerica, MA, USA), containing annexin V phycoerythrin and 7-amino-actinomycin D (7-AAD). Three cell populations can be detected: nonapoptotic live cells (annexin^−^/7-AAD^−^), early apoptotic cells (annexin^+^/7-AAD^−^), and late apoptotic or necrotic cells (annexin^+^/7-AAD^+^). Cells were incubated for 20 min at room temperature in the dark and analyzed by flow cytometry. H_2_O_2_ 300 *μ*M was used as positive control.

### 2.5. Detection of Intracellular ROS

3 or 6 h after CAP treatment, cells were incubated with 2′,7′-dichlorodihydrofluorescein diacetate (H_2_DCFDA, 10 *μ*M) (Sigma-Aldrich, St. Louis, Missouri, USA) for 20 min at 37°C, 5% CO_2_ in the dark. H_2_DCFDA is a nonpolar and nonfluorescent molecule able to diffuse in living cells, where it is hydrolyzed at 2′,7′-diclodihydrofluorescrin (H_2_DCF) by intracellular esterases and trapped into cells. In the presence of ROS, H_2_DCF is oxidized to the fluorescent molecule 2′,7′-diclorofluorescein (DCF), which can be detected by flow cytometry. The fluorescence intensity of DCF is proportional to intracellular ROS levels.

### 2.6. DNA Damage Assays

A complementary experimental approach was used to detect the primary DNA damage (H2A.X histone phosphorylation assay), as opposed to the mutational effects (micronucleus test) that can result from DNA damage.

Phosphorylation of histone p-H2A.X was used as marker of CAP genotoxic potential using FlowCellect™ Histone H2A.X Phosphorylation Assay Kit (Merck Millipore), 6 and 24 h after treatment with CAP. The kit components include the fixation buffer (Part number CS202122) and 1x permeabilization buffer (Part number CS203284), both ready to use. After washing, 1 × 10^6^ cells were suspended in 1 mL fixation buffer and incubated 20 min on ice. After washing with 1x assay buffer, samples were permeabilized using 1 mL permeabilization buffer and incubated for 20 min on ice according to the manufacturer's instructions. After washing, each sample was resuspended in 95 *μ*L of assay buffer and 5 *μ*L of anti-histone H2A.X antibody. The antibody used to detect H2A.X phosphorylated was 20x anti-histone H2A.X-Alexa Fluor® 488 (Part number CS208216). Samples were incubated for 30 minutes in the dark at room temperature. After washing, cells were suspended in 1x assay buffer and analyzed via flow cytometry. Ethyl methanesulphonate (EMS) 240 *μ*g/mL was used as positive control. Some experiments were performed with N-acetylcysteine (NAC) (1 h, 10 mM).

### 2.7. MN Assay

For micronucleus test, Jurkat cells were treated with CAP and incubated for 24 h to allow cell replication. At the end of the incubation, 0.5 × 10^6^ cells were stained according to the manufacturer's instruction of the in Vitro Microflow kit (Litron Laboratories, Rochester, NY, USA). Briefly, cells were first stained with nucleic acid dye A solution (300 *μ*L), containing ethidium monoazide (EMA) that crosses the compromised membrane of apoptotic and necrotic cells. Since EMA needs photoactivation to covalently bind DNA, cells were kept on ice and photoactivated. Complete lysis solution 1 (500 *μ*L) was added to digest the cytoplasmatic membrane and release nuclei and micronuclei (MN). To complete membrane lysis and obtain the complete release of nuclei and MN, cells were then treated with complete lysis solution 2 (500 *μ*L). Both cell lysis solutions contain the dye SYTOX green that labels chromatin. The double staining with EMA and SYTOX green of chromatin allows discriminating nuclei and MN in living cells (SYTOX green^+^) from fragments derived from damaged chromatin of dead/dying cells (EMA^+^/SYOTX green^+^). At the end of the incubation, cells were analyzed by flow cytometry. MN were distinguished from nuclei for their smaller dimension and thus for their lower fluorescence: MN exhibit 1/100th to 1/10th of the intensity of duplicated nuclei [33]. EMS (240 *μ*g/mL) was used as positive control. The gating strategy is carefully described in the protocol of the in Vitro Microflow kit (Litron Laboratories). Using a negative control for setup, samples were acquired adjusting forward scatter (FSC) and side scatter (SSC) voltages to bring nuclei into view. The lower bounds of the region were approximately 2 logs lower in FSC and SSC than the bottom left edge of the nucleus events. Then, EMA fluorescence was set according to discriminate nuclei from healthy and dead cells. The position of the “FSC versus SYTOX” region was adjusted until nuclei were positioned. Much of the chromatin associated with dead/dying cells fell above an appropriately located “FSC versus SYTOX” region. Then, the position of the “SSC versus SYTOX” region was adjusted until nuclei were positioned. Much of the chromatin associated with dead/dying cells fell above an appropriately located “SSC versus SYTOX” region. The final plot gave results on the number of MN and nucleated cells, showing SYTOX green versus FCS. From this test, cell cycle perturbations were recorded by studying fluorescence histograms of SYTOX green, a nucleic acid dye.

To validate the results obtained by in Vitro Microflow kit, the analysis of MN in binucleated cells was performed by adding cytochalasin B (Sigma-Aldrich; final concentration of 6 *μ*g/mL) after 44 h of culture [34]. At the end of the 72 h incubation period, Jurkat cells were fixed with ice-cold methanol/acetic acid (1 : 1). The fixed cells were put directly on slides using a cytospin and stained with May-Grünwald-Giemsa. All slides were coded before scoring. The criteria for scoring micronuclei were as described by Fenech [35]. At least 2000 binucleated lymphocytes were examined per concentration (two cultures per concentration) for the presence of one, two, or more micronuclei.

### 2.8. Analysis of p53, Bax, Bcl-2, and Caspase-8 Protein Levels

24 and 48 h after cell treatment, the analysis of protein levels involved in the apoptotic process was performed. Briefly, 0.5 × 10^6^ cells were fixed using a 4% paraformaldehyde solution in PBS 1x and permeabilized in 90% ice-cold methanol solution. Cells were then incubated with the following antibodies: p53 (1 : 200, Santa Cruz Biotechnology, Dallas, TX, USA), Bax (1 : 200, Santa Cruz Biotechnology), and Alexa Fluor 488-labeled Bcl-2 (1 : 200, BioLegend, San Diego, CA, USA). The cells, except those stained with Bcl-2, were washed, incubated with fluorescein isothiocyanate-labeled secondary antibody (1 : 100, Sigma-Aldrich), and analyzed by flow cytometry. Sulforaphane was used as positive control. Mean fluorescence intensity values were calculated. Nonspecific binding was excluded by isotype-matched negative control (1 : 100, eBioscience, San Diego, CA, USA). The expression of caspase-8 was detected by using leucine-glutamic acid-threonine-aspartic acid (LETD) caspase-8-preferred substrate linked to a fluoromethylketone (FMK) that reacts covalently with the catalytic cysteine residue in the active enzymatic center. 6-Carboxyfluorescein (FAM) was used as fluorescent reporter. 24 and 48 h after CAP treatment, nonpermeabilized cells were stained with 10 *μ*L of freshly prepared 10x working dilution of FAM-LETD-FMK solution (Merck Millipore) and incubated for 1 h at 37°C in the dark. At the end of incubation, cells were washed and suspended in 150 *μ*L of 7-AAD diluted 1 : 200 in 1x working dilution wash buffer (Merck Millipore), incubated for 5 min at room temperature in the dark, and analyzed via flow cytometry. Staurosporine was used as positive control.

### 2.9. Real-Time PCR

Total RNA was isolated using Agilent Total RNA isolation Mini Kit (Agilent Technologies, Santa Clara, CA, USA), according to the manufacturer's instructions. Briefly, 350 *μ*L of lysis buffer was added to cell pellet and the cell homogenate centrifuged through a miniprefiltration column. The eluate was mixed with an equal volume of 70% ethanol solution, incubated for 5 min at room temperature, and centrifuged through a mini-isolation column. The eluate was discharged, and after column washing, the RNA was eluted by adding nuclease-free water and stored at −20°C. cDNA was synthesized using High Capacity cDNA Reverse Transcription Kit (Life Technologies, Carlsbad, CA, USA). Briefly, 200 ng total RNA was added to 10 *μ*L reaction kit mixture with RNAse inhibitor, according to manufacturer's recommendations. Changes in mRNA expression were measured by using TaqMan® gene expression assay (Life Technologies) and Real-Time PCR (Step One, Life Technologies). The variation in the expression of the following genes was analyzed: TP53 (Hs01034349_m1), Bax (Hs00180269_m1), Bcl-2 (Hs00608023_m1), SOD1 (Hs00533490_m1), CAT (Hs00156308_m1), and GSR2 (Hs00167317_m1). 18S ribosomal RNA and actin beta (ACTB) (Hs99999901_s1 and Hs99999903_m1, resp.) were used as endogenous controls. Each measurement was performed in triplicate. Data were analyzed through the 2^−ΔΔCt^ method.

### 2.10. Statistical Analysis

The results are expressed as mean ± SEM of at least 3 independent experiments. Statistical analysis was performed using repeated ANOVA, followed by Bonferroni as posttest, using GraphPad InStat version 5.00 (GraphPad Prism, La Jolla, CA, USA). *P* < 0.05 was considered significant.

## 3. Results

### 3.1. CAP Induces Hydrogen Peroxide and Nitrite Production in Treated Medium

**Figure 1:**
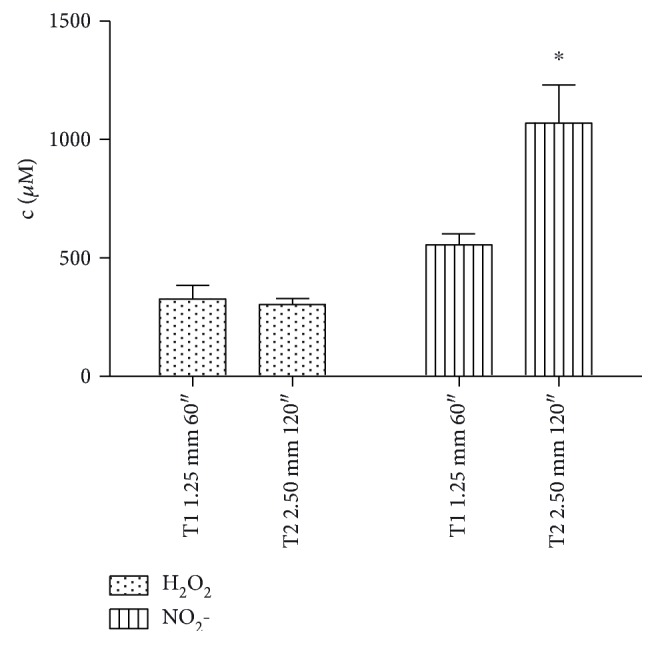
Hydrogen peroxide and nitrite concentration in plasma-treated complete medium, measured by specific colorimetric kits. Shown data are the mean of three different experiments. ^∗^*P* < 0.05 versus T1 nitrite.

Since the effects of plasma on cancer cells seem to be mediated by reactive species, we first studied the ability of CAP to produce H_2_O_2_ and NO_2_- in medium. In Figure 1(), we show the concentrations of hydrogen peroxides and nitrites in 1 mL of complete cell culture medium after plasma treatments, recorded by a specific colorimetric kit. Hydrogen peroxides and nitrites are not present in the untreated medium. Both CAP treatment conditions, T1 and T2, induced the production of similar concentrations of hydrogen peroxides (about 310 *μ*M). On the other hand, nitrites concentration induced by T2 was significantly higher (up to about 1068 *μ*M) compared to the concentration produced by T1 (about 556 *μ*M).

### 3.2. CAP Induces Apoptosis in Jurkat Cells

**Figure 2:**
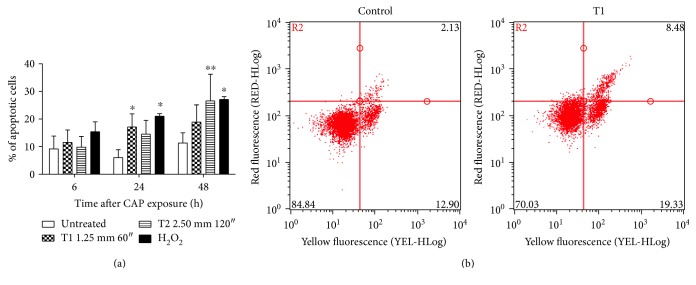
(a) % of apoptotic cells 6, 24, and 48 h after CAP treatment of Jurkat cells under T1 (1.25 mm 60″) and T2 (2.50 mm 120″) conditions (mean of three different experiments). H_2_O_2_ 300 *μ*M was used as positive control. (b) Representative dot plot of annexin V (yellow fluorescence) versus 7-amino-actinomycin D (7AAD, red fluorescence) for the untreated cells and cells 24 h after CAP treatment under T1 condition. ^∗^*P* < 0.05; ^∗∗^*P* < 0.01 versus the untreated cells.

One of the most relevant mechanisms of action of anticancer drugs is the induction of apoptosis. Thus, we investigated the ability of CAP to induce apoptosis by flow cytometry. A significant increase in apoptotic cells was found in samples after treatment under both the tested CAP operating conditions. As an example, 17.1% of apoptotic cells was observed 24 h after CAP exposure at T1 (versus 6.0% of the untreated cells) and 26.6% 48 h after CAP exposure at T2 treatment condition (versus 11.3% of the untreated cells) (Figure 2).

**Figure 3:**
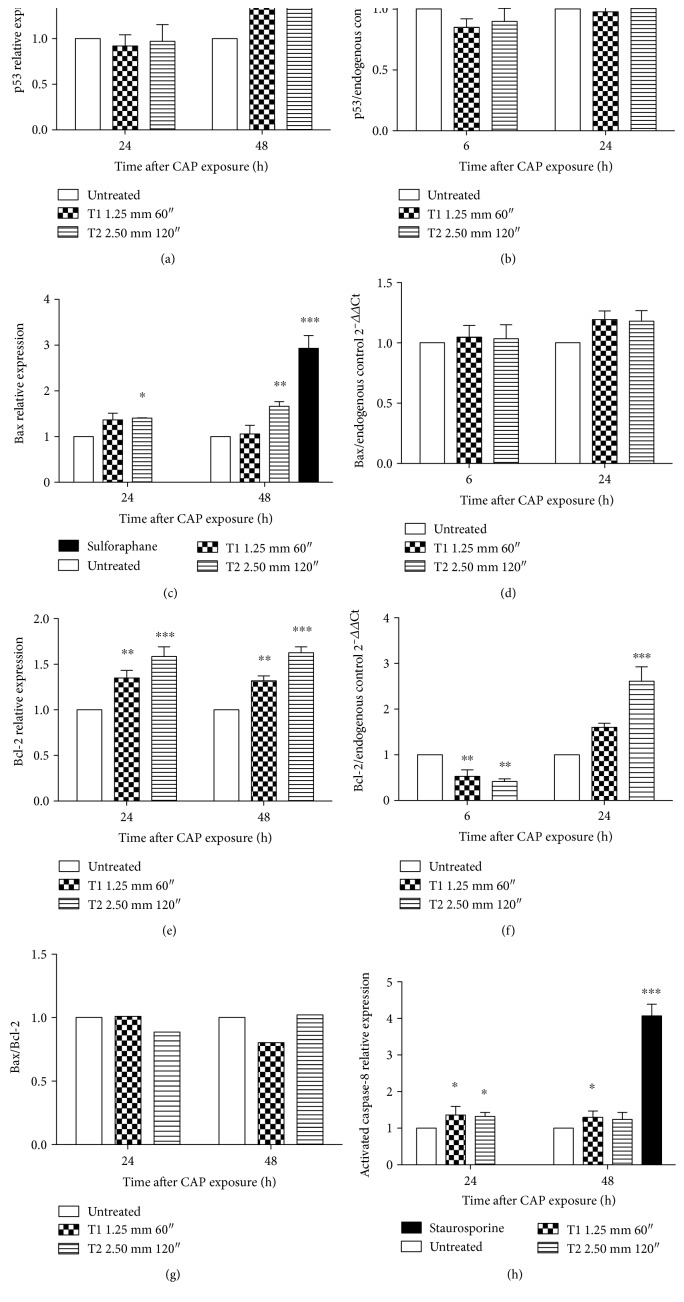
Effects of CAP on gene involved in the apoptotic pathway in Jurkat cells. Relative protein expression of (a) p53, (c) Bax (sulforaphane was used as positive control), and (e) Bcl-2 after 24 and 48 h after CAP exposure. mRNA expression of (b) p53, (d) Bax, and (f) Bcl-2 6 and 24 h after CAP exposure at T1 and T2 conditions. 18S ribosomal RNA and actin beta (ACTB) were used as endogenous controls. (g) Bax/Bcl-2 ratio. (h) Relative expression of caspase-8 24 and 48 h after CAP treatment. Staurosporine was used as positive control. Data are the mean of at least three different experiments. ^∗^*P* < 0.05; ^∗∗^*P* < 0.01; ^∗∗∗^*P* < 0.001 versus the untreated cells.

To evaluate the apoptotic mechanisms evoked by CAP, the expression of key genes involved in the apoptotic pathway was analyzed. CAP increased p53 protein expression 48 h after treatment. p53 expression in cells treated under condition T2 resulted in a 1.74-fold increase compared to the untreated cells, whose expression was normalized to 1 (Figure 3(a)). No modulation for p53 was observed at RNA level for all tested treatment conditions (Figure 3(b)).

To explore the involvement of the intrinsic and/or extrinsic pathway in CAP-induced apoptosis, the expression of Bax/Bcl-2 and caspase-8 was analyzed. An increase in Bax and Bcl-2 expression was observed, with the highest effect in cells treated under the T2 condition and 48 h after CAP exposure. Bax and Bcl-2 expression resulted 1.66-fold and 1.63-fold higher, respectively, than the untreated cells (Figures 3(c) and 3(e)). Sulforaphane, used as positive control, increased the expression of Bax by about 3 times after 48 h treatment versus the untreated cells. Of note, the Bax/Bcl-2 ratio was not significantly changed following CAP treatment (Figure 3(g)). Any variation was observed for RNA expression of Bax neither 6 nor 24 h after CAP treatment (Figure 3(d)). The modulation of Bcl-2 by CAP treatment was more complex. Bcl-2 was downregulated 6 h after treatment using both the operating conditions but upregulated 24 h after CAP treatment (Figure 3(f)). The expression of caspase-8 was significantly increased both 24 and 48 h after CAP treatment and in both conditions. In particular, after 24 h T1 CAP treatment, the expression was 1.36-fold higher than the untreated cells (Figure 3(h)). Staurosporine, used as positive control for caspase-8, induced a 4-fold increase after 48 h of treatment, compared to the untreated cells.

### 3.3. CAP Increases Intracellular ROS Levels and Modulates the Oxidative Stress Pathway

**Figure 4:**
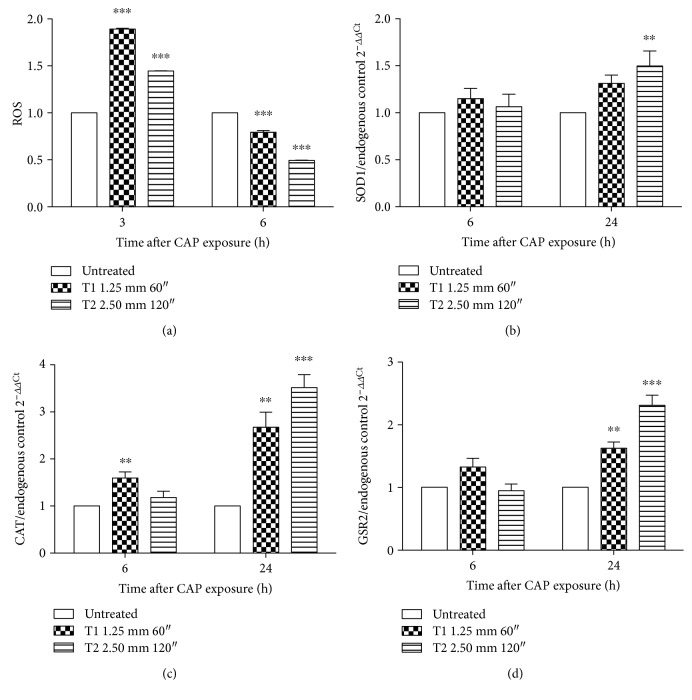
Effect of CAP on ROS levels and antioxidant enzymes. (a) Intracellular levels of ROS in Jurkat cells after CAP exposure for 3 and 6 h, measured via 2′,7′-diclorofluorescein (DCF) fluorescence. Relative mRNA expression of (b) SOD1, (c) CAT, and (d) GSR2 6 and 24 h after CAP treatment. 18S ribosomal RNA and actin beta (ACTB) were used as endogenous controls. Data are the mean of three different experiments. ^∗∗^*P* < 0.01; ^∗∗∗^*P* < 0.001 versus the untreated cells.

We previously demonstrated that CAP produces H_2_O_2_ and NO_2_- in medium. For this reason, we studied the effects of CAP on the modulation of intracellular ROS levels. The cytofluorimetric measurement of 2′,7′-diclorofluorescein (DCF) indicated a significant increase in intracellular ROS levels after CAP treatment. In particular, 3 h after CAP exposure, ROS levels were 1.89-fold and 1.45-fold higher in cells treated at T1 and T2 operative conditions, respectively, compared to control (Figure 4(a)). However, 6 h after CAP treatment, a significant reduction of intracellular ROS levels was observed in both the investigated cases (Figure 4(a)). Thus, the ability of CAP treatment to modulate the oxidative stress pathway was analyzed. A posttranscriptional modulation of SOD1 was observed, as indicated by the 1.5-fold higher expression than the untreated cells 24 h after treatment at the T2 condition (Figure 4(b)). An upregulation of CAT and GSR2 was also detected after CAP exposure. The highest effect was observed for both genes 24 h after the T2 CAP treatment (3.52- and 2.31-fold higher than the untreated cells, resp.) (Figures 4(c) and 4(d)).

### 3.4. CAP Has a Genotoxic and Mutagenic Effect

**Figure 5:**
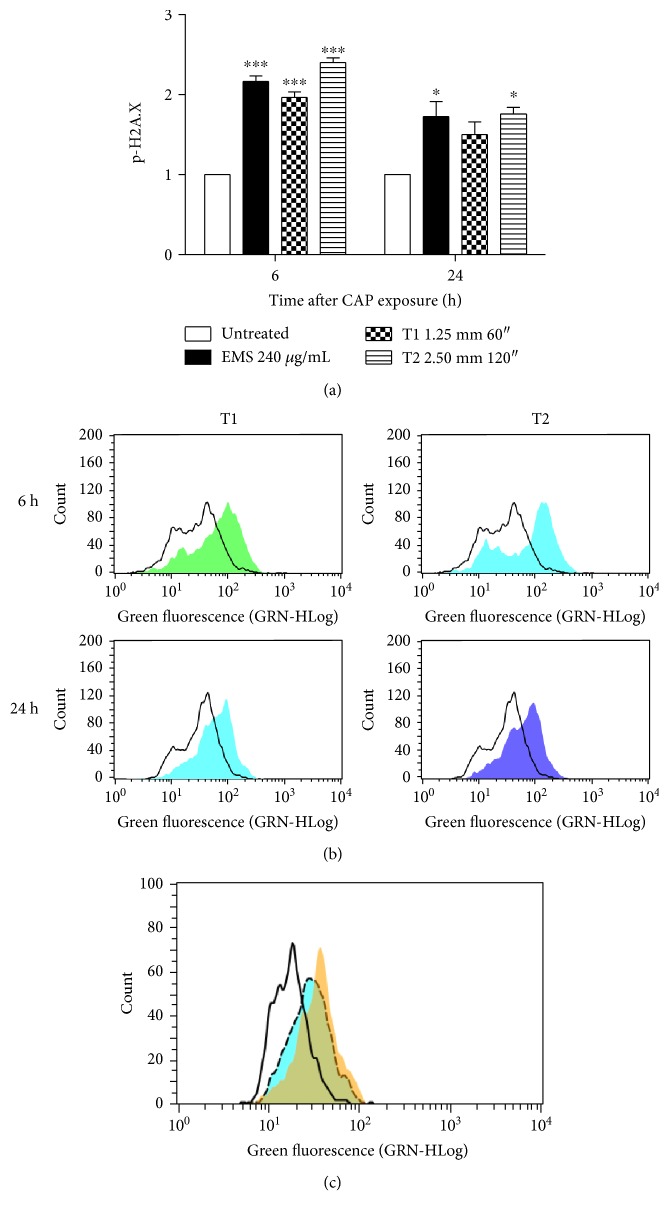
(a) Relative expression of p-H2A.X in Jurkat cells 6 and 24 h after CAP exposure. Ethyl methanesulfonate (EMS) was used as positive control. Data are the mean of three different experiments. (b) Representative histograms of p-H2A.X expression after 6 and 24 h at T1 and T2 treatment conditions. (c) Histogram of p-H2A.X expression representative of three different experiments performed with similar results. Black line: untreated cells; yellow histogram: cells after T1 CAP treatment; dashed line: cells pretreated with N-acetylcysteine (NAC) 10 mM and then exposed to CAP. ^∗^*P* < 0.05; ^∗∗∗^*P* < 0.001 versus the untreated cells.

Since we demonstrated that CAP is able to generate RONS, its genotoxic potential should be carefully examined. For this reason, in the final part of our study, we used different tests of genotoxicity with the aim to detect the net and actual mutagenic effect of the plethora of lesions caused by CAP and to directly relate the DNA damage to the mutagenic effect. To investigate whether CAP was able to induce DNA damage, the phosphorylation of H2A.X (p-H2A.X) was analyzed. 6 h after CAP exposure, a 2.16-fold increase in p-H2A.X compared to the untreated cells was observed. 24 h after CAP treatment, the level of p-H2A.X was significant only under the T2 condition (Figures 5(a) and 5(b)). In order to assess the role of ROS on p-H2A.X assay, we pretreated cells with N-acetylcysteine (NAC) 10 mM for 1 h and then with CAP. After 6 h from treatment, we observed a decrease in p-H2A.X in cells pretreated with NAC and then exposed to CAP compared to cells treated only with CAP at the T1 condition (1.61-fold versus 1.9-fold compared to the untreated cells) (Figure 5(c)).

**Figure 6:**
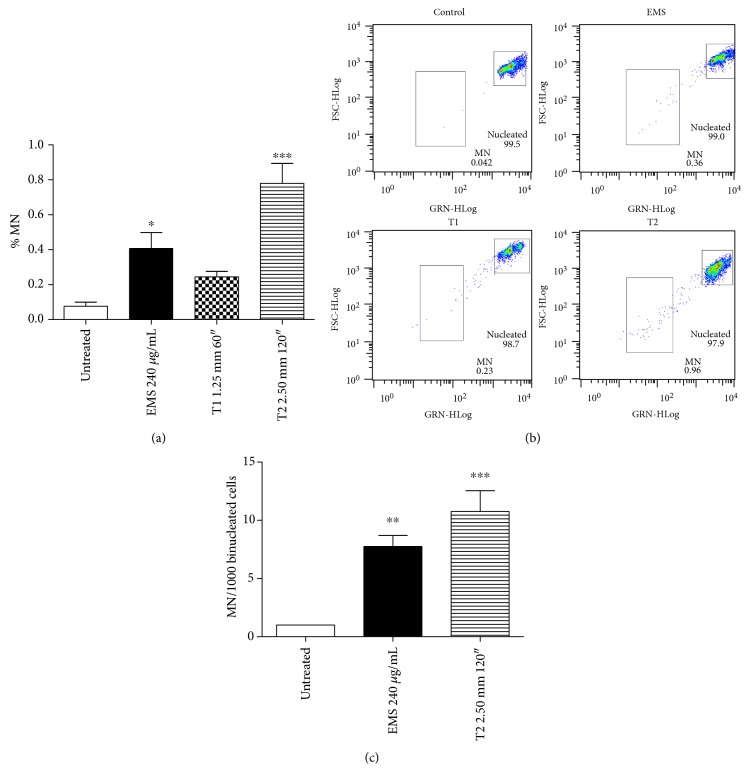
Induction of micronuclei (MN) 24 h after CAP exposure in Jurkat cells. Ethyl methanesulfonate (EMS) was used as positive control. (a) % of MN recorded flow cytometrically by SYTOX green/EMA. Data are the mean of three different experiments. (b) Representative results of the final gate of SYTOX fluorescence (GRN) versus forward scatter (FSC) of control, EMS, and CAP treatments under T1 and T2 conditions. (c) MN recorded microscopically on binucleated cells. ^∗^*P* < 0.05; ^∗∗^*P* < 0.01; and ^∗∗∗^*P* < 0.001 versus the untreated cells.

To understand whether CAP causes retained alterations in DNA sequence and thus a mutagenic effect, the generation of micronuclei (MN) was analyzed. 24 h after CAP exposure, a 3.2-fold increase in the frequency of MN was observed at the T1 condition (MN 0.24% versus 0.08% of the untreated cells) and a 10.2-fold increase at the T2 (MN 0.78%), compared to the untreated cells. The increase in MN frequency induced by EMS, used as positive control, was 5.3-fold compared to the untreated cultures (Figures 6(a) and 6(b)). The correlation index between the p-H2A.X expression and the MN frequency calculated by GraphPad InStat version 5.00 at 24 h posttreatment was 0.79. The 0.79 value indicates a strong positive linear relationship between the genotoxicity and mutagenicity of CAP. The MN test performed on binucleated cells by microscopic analysis confirmed the mutagenic effect of CAP (Figure 6(c)).

By analyzing the fluorescence histograms of SYTOX green, a nucleic acid dye, we demonstrated that the treatment of Jurkat cells with CAP inhibited cell-cycle progression and induced an accumulation of cells in the G2/M phase. For example, both CAP treatments under T1 and T2 conditions increased the percentage of cells in G2/M phase (69% and 72%, resp., versus 40% of the untreated cells), with a compensatory decrease of cells in S phase (6% and 9%, resp., versus 22% of control) and G0/G1 phase (25% and 19%, resp., versus 38% of the untreated cells).

## 4. Discussion

The effects of CAP on cancer cells were observed in an *in vitro* leukemia model. Complete medium containing serum was treated by means of DBD with two different operative conditions using different treatment time and gap that are known to play a crucial role in the RONS generation, as reported by Kuchenbecker et al. [36]. The plasma treatments induced the production of similar concentrations of hydrogen peroxide and higher concentration of nitrite in the T2 condition (120″). This difference on RONS concentrations could be related to the presence of serum in the treated medium that is known to scavenge hydrogen peroxide [24] while not affecting the nitrite accumulation. Furthermore, we hypothesize that the increase in nitrite concentration after T2 treatment could be ascribed to the higher gap (2.50 mm), which enhances the interaction between plasma and nitrogen in air.

The observed overexpression of p53 indicates the contribution of this protein to CAP-induced apoptosis. Apoptosis could be mediated by mitochondrial/intrinsic pathway or receptor/extrinsic pathway [37]. p53, a crucial protein in both mechanisms, has been identified as one of the most targetable molecules for developing anticancer treatment. Its activation, usually in response to DNA damage, and relevant signaling are key steps to induce apoptosis [38]. Apoptosis is a defense mechanism against stressed, damaged, and/or stimulated cells, and different regulatory pathways are involved to orchestrate this mechanism of cell death [37]. The involvement of the intrinsic pathway on CAP-induced apoptosis was verified through the analysis of Bax and Bcl-2 expression. Bax and Bcl-2 are two of the major proteins of Bcl-2 family and their ratio, meaning the balance of the expression between pro- and anti-apoptotic pathways determines apoptosis execution in response to external stimuli, thus, cell fate [39, 40]. The protein expression of Bax resulted significantly upregulated after CAP treatment, but no modulation was observed at RNA level. Moreover, CAP provoked an upregulation of Bcl-2 at protein level, but the effect was different at RNA level. In fact, mRNA expression was downregulated 6 h after treatment and upregulated longer time (i.e., 24 h) after CAP treatment. To understand how cells respond to stress, we quantified both protein and RNA expression. The different modulation of Bax and Bcl-2 at mRNA and protein levels recorded in our study could be due to the tiny mechanisms involved in the posttranscriptional regulation to turn mRNA into protein and the different time of proteins' turnover [41]. The observed increase in Bcl-2 protein observed in our experimental setting may also serve as a compensatory protection of Jurkat cells upon CAP insult. The increase in Bcl-2 expression could be justified by its involvement in the modulation of cellular oxidative stress, beside its well-established role as antiapoptotic protein [42]. In fact, it has been demonstrated that Bcl-2-overexpressing cells show a significant, but subpathological, enhancement of ROS output that, in turn, stimulates the antioxidant defense. Furthermore, the different regulations of Bcl-2 mRNA at different time points may be due to the different mechanisms of compensation triggered by stress or adaptation signaling in cancer cells, such as epigenetic mechanisms [43]. Other studies showed how CAP treatment reduced the mitochondrial membrane potential, downregulated the expression of Bcl-2 that, in our experimental system, we observed at RNA level, activated PARP, and released apoptosis-inducing factor from the mitochondria [13]. However, some limitations in our study deserve a consideration. The activation of the proapoptotic function of Bax can be regulated by interdependent mechanisms including posttranslational modifications like phosphorylation. Protein kinase C*ζ* plays a critical role in promoting cell survival. It may phosphorylate and interact with Bax. Through these mechanisms, it leads to sequestration of Bax in cytoplasm and abrogation of its proapoptotic function [44]. We did not analyze the phosphorylation status of Bax with regard to protein and mRNA expression and its role in the modulation of apoptosis induced in cells treated with CAP.

The significant increase in caspase-8 activation and p53 upregulation indicates the involvement of the extrinsic pathway by CAP treatment. The cross talk between extrinsic and intrinsic pathways of apoptosis is regulated by Bid, a proapoptotic member of Bcl-2 family. The cleavage of Bid is mediated by caspase-8, which induces apoptosis by releasing cytochrome c from the mitochondria [37]. Other studies on DBD plasma demonstrated that the increased transcription of Bax and caspase-8 on U937 (human monocytic lymphoma) cells contributes to plasma-induced apoptosis [45].

Our results are in the agreement with previous studies on the effects of plasma on Jurkat cells. Bundscherer and colleagues investigated the effects of nonthermal plasma, using the kinpen, in Jurkat cells and demonstrated an increase in apoptotic events, depending on plasma treatment time. They confirmed the proapoptotic effect of plasma, which is due to the activation of caspase-3 and MAPK signaling pathway [46]. Cell death induced by CAP on Jurkat cells was also reported using a volume barrier discharge device in which the target is located between the two electrodes. No effects were observed when low energy (128 J/cm^2^) was used to generate plasma discharge, but an apoptotic pattern was registered for medium energy (255 J/cm^2^) and a purely necrotic pattern for high energy (425 J/cm^2^) [47]. In our experimental system, a significant increase in necrotic events was observed when cells were treated with DBD for longer time (i.e., 120″) at the shortest distance from the medium (1.25 mm) after 24 h from treatment.

Oxidative stress has a major role in many biological processes [48]. The increase in ROS levels is involved in different physiological processes, such as proliferation and differentiation, while, over a certain intracellular level, ROS are responsible for cytotoxic and cytostatic effects [49]. Targeting oxidative equilibrium of tumor cells is currently a recognized approach to kill cancer cells [50, 51]. In fact, tumor cells are usually characterized by an increased basal oxidative stress, making them vulnerable to chemotherapeutic agents that further enhance ROS levels [52]. RONS generation has been actually proposed as a new theory behind CAP selectivity [53]. We observed a significant increase in intracellular ROS levels after CAP treatment, according to literature data [23, 54], hence, potentially leading to a selective death of tumor cells. In our experimental settings, CAP modulated the intracellular ROS levels 3 h after treatment, whereas 6 h after plasma treatment, ROS were restored at levels similar or even lower than those of the pretreatment condition. This may be probably due to cellular compensatory mechanisms [55], such as the observed posttranscriptional regulation of SOD1, CAT, and GSR2. The major cellular defense against O_2_^•−^ and peroxynitrite is a group of oxidoreductases known as SODs, which catalyze the dismutation of O_2_^•−^ into oxygen and H_2_O_2_ that is detoxified to water by catalases and peroxidases [56]. GSH and GSH-dependent enzymes including GSR play a key role in cellular detoxification processes that enable organism to cope with various internal and environmental stressors [57]. The antioxidant response is therefore orchestrated from different enzymes that together in combined action give the antioxidant effect. The observed upregulation of SOD1, CAT, and GSR2 enzymes at RNA level supports the hypothesis that the combined interaction of plasma constituents, electric field, ions, and electrons with the biological cellular components induces oxidative stress.

However, the detailed biological mechanisms by which CAP can induce apoptosis is not yet clear [58]. As previously mentioned, CAP affects cells both via direct and indirect treatments. In particular, H_2_O_2_-cooperating active species seem to be responsible to induce membrane alteration, increasing the permeability to other extracellular reactive species and further modulating intracellular ROS levels [13].

Due to the capacity of CAP to generate RONS, we evaluated its genotoxic potential through the analysis of premutational and mutational events. The phosphorylation of H2A.X is a reliable marker of genotoxicity and useful to predict the genotoxic potential of a compound [59]. In our study, CAP treatment induced a significant increase in the level of p-H2A.X under all tested treatment conditions, with higher values at 6 h as compared to 24 h after plasma treatment. Notably, the phosphorylation assay is a clear index of the ability of a xenobiotic, or in this case, a physical treatment, to interact with the DNA, thus causing a premutational lesion that could still be repaired by the DNA repair systems [60]. Furthermore, the decrease in DNA damage recorded 24 h after CAP exposure could be justified by the decrease in intracellular ROS levels observed at the same time point [61]. The reduction in p-H2A.X expression recorded after pretreatment of cells with NAC suggests that intracellular ROS levels play a role in the DNA damage induced by CAP. The evidence that scavenging proteins limited the effect of nonthermal plasma was previously reported. For example, Ma and colleagues showed that the cytotoxic effect of CAP is dose dependently reduced by the presence of ROS scavengers [23]. However, in our experimental settings, NAC did not fully abolish H2A.X phosphorylation. This means that other CAP components may interact with DNA. Furthermore, a growing body of evidence demonstrated that p-H2A.X is always induced when DNA double-strand breaks (DSBs) are provoked, but p-H2A.X should not be considered an unequivocal marker of DSBs and could not be a participant in the DNA damage response [62, 63]. To understand whether the CAP-induced DNA lesions are turned into mutations, the MN test was performed. To the best of our knowledge, this is the first report describing that CAP exposure significantly increased the frequency of MN on Jurkat cells at both exposure conditions, thus indicating a mutagenic and irreversible effect for CAP. The recovery of damaged cells under conditions where cell death occurred by apoptosis [64] suggests that the destruction of genetically damaged cells by the apoptosis effector pathways is not completely efficient. A huge amount of data demonstrated the genotoxic effects of plasma treatments in both prokaryotic and eukaryotic systems, but due to the complexity of DNA-plasma interaction, sometimes conflicting results are reported. Some studies showed the lack of mutagenic effect of plasma. For example, the treatment with the argon plasma jet kinpen did not display a mutagenic potential [65–67]. Taking into account the heterogeneity of plasma composition, each plasma source should be tested for both efficacy and safety aspects [24].

In conclusion, our results provide a deeper understanding of the potential of CAP as a promising physic-pharmacologic strategy in the oncological field and pose the basis for the evaluation of its toxicological profile.
